# Digital patient-reported outcome measures in palliative home care: A feasibility study

**DOI:** 10.1177/02692163251409294

**Published:** 2026-02-05

**Authors:** Katerina Hriskova, Isabel Sophie Burner-Fritsch, Farina Hodiamont, Anna Bolzani, Stefanie Kolmhuber, Christina Ramsenthaler, Claudia Bausewein

**Affiliations:** 1 Department of Palliative Medicine, LMU University Hospital, LMU Munich, Munich, Germany; 2 Department of Health Sciences, Institute of Nursing, ZHAW Zurich University of Applied Sciences, Winterthur, Switzerland

**Keywords:** patient reported outcome measures, digital health, home care services, pilot projects, feasibility studies

## Abstract

**Background:**

Patient-reported outcome measurement supports high quality patient-centred palliative care. Little is known about whether their digital application is feasible in palliative home care.

**Aim:**

To test the feasibility of digital patient-reported outcome measure (ePROM) in specialist palliative home care (SPHC)

**Design:**

A feasibility study employing a mixed-methods design (Palli-MONITOR Phase II). The tested ePROM intervention was based on the electronic version of the Integrated Palliative Care Outcome Scale (eIPOS). Data collection included the recruitment and drop-out rates, ePROM user characteristics and information on technical feasibility, and focus groups with SPHC professionals. Descriptive statistics were used to analyse the quantitative data, while focus groups were analysed using the framework approach. Integrated analysis was conducted through joint display.

**Setting/participants:**

Four German SPHC teams; patients used personal devices to complete eIPOS, with data sent to the SPHC electronic medical record; professionals joined focus groups.

**Results:**

The overall recruitment rate was 4.7% (82/1744), and 22.7% (82/361) among eligible patients. 60/82 patients completed the study. A total of 470 eIPOS forms were submitted to the SPHC teams. The rate of non-responses for closed-ended IPOS-items was low (max. 5.3%). Professionals noted that recruitment was challenged by patients’ unstable conditions, short care duration, time constraints, team attitudes and technical barriers like limited internet access or device unfamiliarity.

**Conclusion:**

Not all patients in SPHC can use ePROMs due to limited life expectancy and technical barriers. However, consistent and complete use of eIPOS forms indicates that it is feasible for digitally literate patients and can effectively support care.

**What is already known about the topic?**

- There is a paucity of evidence on the feasibility of incorporating digital patient-reported outcome measurement into the clinical routine of German palliative home care.
- The development of a digital patient-reported outcome measurement system for palliative home care is fraught with challenges, including but not limited to the patient’s poor general condition and lack of internet access or limited familiarity with digital tools.
- Specialist palliative home care professionals state that the system for digital patient-reported outcome measurement tools must align with the organisational structure of the service.

**What this paper adds?**

- Key challenges on the feasibility of electronic patient-reported outcome measurement in specialist palliative home care are patients’ fluctuating symptom burden, technical issues and care giver influence.
- As a digital assessment tool, IPOS is feasible and acceptable.
- Which patients in specialist palliative home care are able to use digital PROMs.

**Implications for practice, theory or policy**

- Digital PROM is feasible for some of the patients in specialist palliative home care.
- The introduction of digital PROMs should start earlier in the palliative care process.
- Providing easy-to-use, application-based ePROM platforms and ensuring robust technical support could reduce barriers to the use of ePROMs.

## Background

Patient-centredness is increasingly important in health care and also palliative care including key characteristics like encouraging patient participation, ensuring empowerment and shared decision-making.^1^ Furthermore, patient-reported outcomes illustrate the patient perspective.^2^ Research suggests positive effects of the measurement of patient-reported outcomes (PROM), such as improving symptom recognition, supporting efficient and effective care, improving emotional and psychological outcomes for patients, and improving patient empowerment.^3–8^ The implementation of digital PROMs offers advantages over the use of paper-based versions: The time and organisation required by patients is reduced^9,10^ and they can review their own history of reported information.^11^ A digital data flow with immediate availability of information could overcome the barriers of paper-based PROM use, especially in palliative home care.^7,12,13^ Despite the positive effects, it is questioned whether palliative patients can use PROMs independently, especially in the context of a digital implementation.^14^ Given the benefits of electronic PROM (ePROM) and their unexplored use in palliative home care in Germany, we conducted the Palli-MONITOR project (Monitoring of palliative care needs in specialist palliative home care using an electronic version of the Integrated Palliative care Outcome Scale (clinical trials NCT03879668), 10/18-03/22).^15^ The overall study followed the MRC framework for the development of complex interventions. The Intervention eIPOS was developed in Phase I of the Palli-MONITOR study.^16,17^ The aim of the phase II was to test the feasibility of a digital patient-reported-outcome measure (eIPOS) in the specialist palliative home care setting. Objectives were to evaluate the feasibility of eIPOS use in terms of participant recruitment, participant characteristics, data completion, usage rate and technical practicability of the planned intervention.^15^ The professional perspective on the implementation of eIPOS in specialist palliative home care setting was reported elsewhere.^16^

## Materials and methods

### Study design

This study was conceived as a feasibility study to evaluate the usability of eIPOS within the German specialist home care setting. Our understanding of feasibility studies aligns with the definition provided by Bond et al.,^18^ which encompasses considerations of whether something will work, can be done, should proceed with it, and, if so, how. While the primary objective was to assess feasibility, we adopted a convergent mixed-methods approach, as outlined in the taxonomy by Creswell and Plano Clark,^19^ to test the feasibility and gain deeper insights into the factors influencing the feasibility of the eIPOS intervention. Quantitative data were obtained from patient screening, inclusion and drop-out rates, as well as the completeness of submitted eIPOS forms. In addition to these data, a focus group was conducted with study nurses and professionals from the participating facilities. The structure of this article follows the CONSORT 2010 checklist for reporting pilot or feasibility trials (CONSORT 2010 extension for pilot and feasibility trials checklist). As recommended by Lancaster et al.,^20^ this checklist is also suitable for reporting non- randomised pilot and feasibility studies focusing solely on the intervention arm, with non-relevant items excluded. By reporting only on the feasibility of eIPOS use in this paper, we deviate from the study design planned in the protocol.^15^ Originally, we intended a quasi-experimental design with two control groups. However, when we entered the field, we realised that the clinical documentation of the specialist palliative home care teams was not what we had expected, as standardised symptom measurements were not being carried out on a regular basis. Therefore, the data for the control groups was not valid and reliable for comparison and the following aims listed in the protocol could not be achieved: “To analyse the presumed change in care processes before and after the implementation of eIPOS.” Consequently, this report focuses only on the intervention arm, the eIPOS users. The above-mentioned aim to test the feasibility of the intervention was not affected by the protocol deviations.

### Participants

Participating specialist palliative home care teams: Our study tested the feasibility of eIPOS in four German specialist palliative home care teams, located in rural (*n* = 2) or urban (*n* = 2) areas. In Germany, specialist palliative home care is provided by multi-professional teams throughout the country. This holistic and patient-centred care in the community is available for patients suffering from advanced disease and complex symptom burden.^21^ The services provide various levels of care (advice, coordination, partial care, full care, according to patients’ degree of palliative care needs, with the higher level including a 24/7 call system.^21,22^ Participating patients: Inclusion criteria for eIPOS patients were: (i) receiving care from one of the participating home care teams (including 24/7 on call); (ii) being at least 18 years of age; (iii) able to provide written informed consent; (iv) not too burdened or ill to participate in the study (assessment by clinical team); and fluent in German. A study nurse, in collaboration with the medical staff, screened all patients and contacted eligible patients to inform them about the study. If patients were interested, the study nurse conducted the initial visit to obtain written informed consent and introduced patients to the use of eIPOS. Study nurses were located in each study centre and assisted the team and patients when technical problems or questions arose. Participants in the focus groups: A focus group was conducted with study nurses from the specialist palliative home care teams to explore the recruitment process. In addition, two focus groups with specialist palliative home care professionals were held to examine the acceptability of ePROM, these results are reported elsewhere.^16^ However, as these discussions also provided valuable insights into the feasibility of the intervention, the data were also included in this part of the study. In short, the semi structured discussions were conducted in Spring 2021 online via zoom and moderated by two researchers (IBF, KH), while one researcher provided technical support (SK). Focus group participation was possible for professionals working in the study centres and fluent in German. The topic guide covered issues around the use of eIPOS in specialist palliative home care. The focus groups were recorded on audio and information that did not occur in the audio was saved in postscripts written by one researcher (IBF). The records were transcribed verbatim by an external typing office. The transcription was anonymised.

### Intervention

The Integrated Palliative care Outcome Scale (IPOS) is a valid and reliable holistic measure containing palliative patients’ main concerns, common symptom burden, patient/family distress, existential well-being, sharing feelings with family or friends, information received, and practical concerns, in 17 items.^23,24^ The final IPOS question asked how the questionnaire was completed. As part of the Palli-MONITOR project, the electronic version of IPOS was validated^17^ and implemented in the clinical care of four specialist palliative home care teams that participated in the overall study. Each participating specialist palliative home care team recruited patients over a 12-month period between December 2019 and August 2021. The exact start and end dates varied by team. A 3-month recruitment pause was necessary during the COVID-19 pandemic. All patients receiving care from the participating teams during their respective recruitment periods were screened. Participating patients were invited to use eIPOS on their own internet-enabled device for approximately 2 weeks at an individualised frequency. At the patient’s request, the eIPOS could also be used for a further 2 weeks. Patients using eIPOS received a link via email and then had to log in with their username and password. Palliative symptom burden and problems as measured on eIPOS were transmitted and timely presented in the electronic patient record of the teams, and were used to inform care. Details of the use of eIPOS reported symptoms and problems in care routine are presented in a different publication (trial registration: ClinicalTrials.gov NCT03879668).^15,16^

### Outcomes

1. Recruitment and drop-out rates: They were the primary outcome of interests. The study nurses noted the reasons for ineligibility and non-participation in the study, which allowed us to calculate recruitment rates and the proportion of patients who did not meet the inclusion criteria or who refused to participate. Drop-outs were defined as patients who gave informed consent but withdrew from the study before reaching the required minimum use of eIPOS for 2 weeks. The study nurse contacted the patients or their families to ascertain the reason for the dropout from the study.
2. Characteristics of patients using eIPOS: The study nurse recorded sociodemographic data, medical diagnoses, palliative phase, and Australian Karnofsky performance status on admission from the electronic medical record, after the patients had provided their consent to participate in the study. The Australian Karnofsky Performance Status is a scale that can be used to assess the functional status regarding activity, self-care and independence.^25^ Classification of the palliative phase as stable, unstable, deteriorating, dying and bereaved helps to assess the clinical situation of patients and their families and their care needs.^26,27^ The number and proportion of patients who survived the study period of 2 or 4 weeks was also reported.
3. Technical feasibility: We defined technical feasibility of the intervention by the number of submitted eIPOS forms and their completeness. A higher number of submitted forms and fewer missing items suggest that patients were technically capable of engaging with the tool. Therefore, we report the number of missing/not reported IPOS items. At the initial visit, patients were also asked about their preferred frequency for receiving the email containing the link to eIPOS. The actual number and content of the submitted eIPOS was documented and the data were extracted in anonymised form from the electronic patient record by the study nurses. Comparing the preferred weekly frequency of eIPOS completion with the actual number of eIPOS forms submitted helps to assess whether the digital tool was feasible for patients and whether their expectations of usability matched their actual experience.
4. Feasibility in terms of understanding the recruitment process for the intervention: Focus groups with professionals provided insights into the feasibility of the intervention, particularly in relation to the recruitment process.

### Sample size

The sample size calculation for the intervention group was detailed in the study protocol of the overall Palli-MONITOR project.^15^ For this study, we aimed to include 213 patients.

### Analysis

Quantitative data: Participants’ sociodemographic information, diagnoses, Australian Karnofsky Performance Status and Palliative Care Phase were reported as absolute numbers and percentages. The variables were summarised using descriptive statistics. Categorical variables were summarised using counts and proportions, while continuous variables were summarised using means and standard deviations (SDs) or median and range. The completion of eIPOS was evaluated for each item based on all questionnaires received and reported as a proportion. SAS Version 9.4 was used for all quantitative analyses. Qualitative data: With the focus group transcripts, we conducted a thematic analysis using the framework approach established by Ritchie & Lewis, supported by MAXQDA ©v.2022.1.^28^ A primary analysis was performed on data from focus group with study nurses, while a secondary analysis was performed on data from focus groups with professionals. Inductive codes derived from the data that informed the thematic framework, aligning with the study’s feasibility objectives. The framework development was guided by regular research team meetings. More details on the collection and analysis of focus groups with professionals are provided elsewhere.^16^

Integrated analysis: Data were analysed in two phases with quantitative and qualitative data analysis was conducted separately, followed by an integrated analysis triangulating quantitative and qualitative results. For the integrated analysis of the results, we developed a joint display in a circle format that combined complementary findings.^29^ The triangulation aimed to examine and present whether different data sources contradicted or confirmed each other, or even provide an extension to gain a deeper understanding of the feasibility of ePROM in specialist palliative home care.

### Ethics

The individual study parts (eIPOS-intervention, focus groups) were approved by the local research ethics committee of the Medical Faculty of Ludwig-Maximilians-University Munich, Germany (19-586, 19-585).

## Results

### Recruitment and participant flow

**Figure 1.**
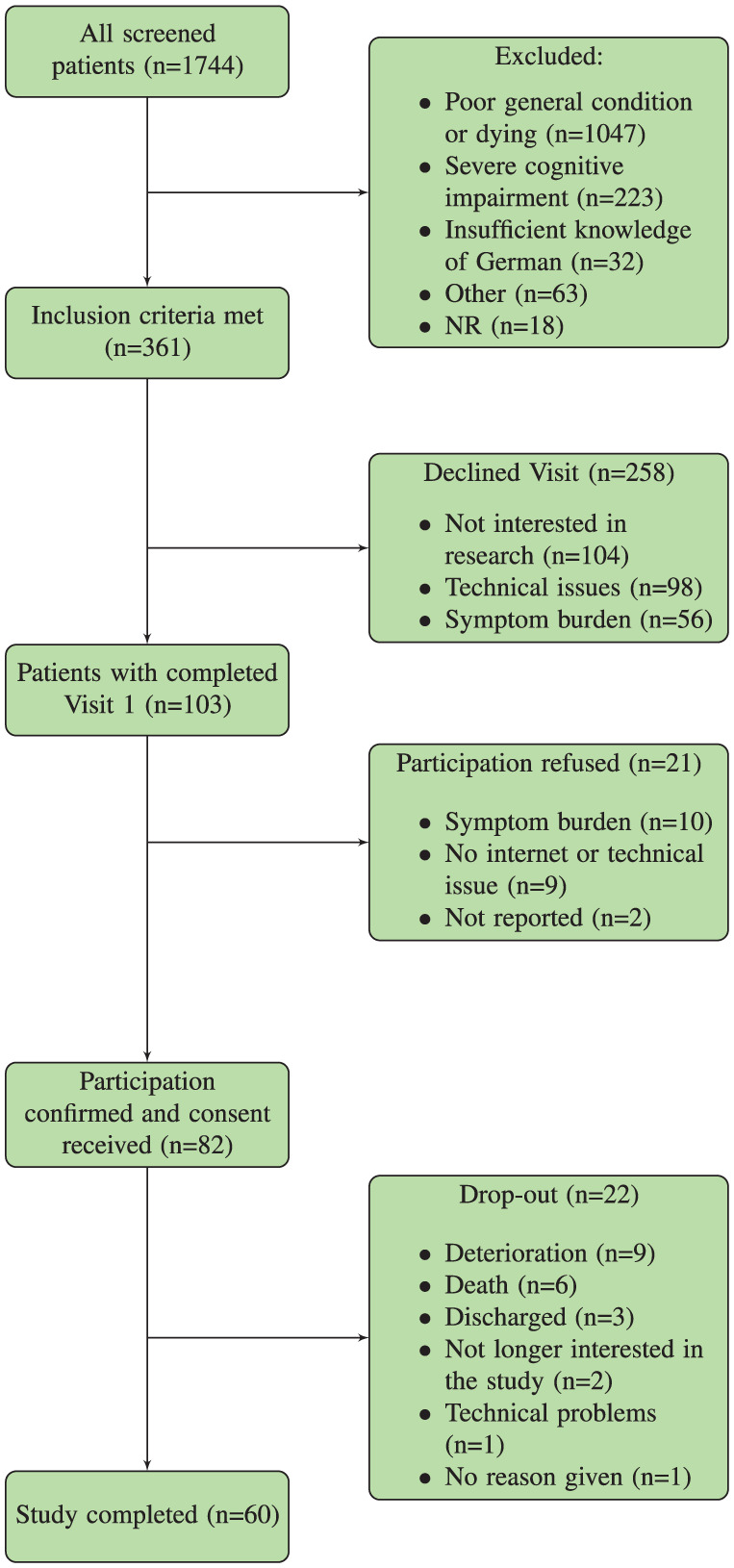
Recruitment flowchart.

During the recruitment period 1744 patients were screened in the four participating teams. Poor general condition was the main reason for exclusion of patients after screening by nurses and professionals. All eligible patients (*n* = 361) were contacted to participate in the study. The study nurse was able to arrange an initial visit for 103 patients. However, at the initial visit, 21 patients refused to provide consent due to high symptom burden or technical difficulties. 82 patients provided written informed consent. 22/82 (28%) patients dropped out of the study prematurely. The main reasons were sudden deterioration in health (*n* = 9), death (*n* = 6), discharge (*n* = 3), not interested in the study anymore (*n* = 2), and technical problems (*n* = 1). One patient did not provide a reason. Thus, the overall recruitment rate corresponded to 4.7%. Taking into account only those patients who met the inclusion criteria (*n* = 361), the recruitment rate was 22.7%. For more details, see Figure 1.

### Characteristics of patients using eIPOS

In total, 40 women and 42 men used the eIPOS. The mean age of eIPOS participants was 68.5 years. As shown in Table 1, 87% had an oncological disease as main diagnosis. Other leading diagnoses were cardiac and respiratory diseases. At the time of enrolment, palliative care phases were almost equally distributed, no patient was in the terminal phase.

**Table 1.** Characteristics of the eIPOS patients at the time of inclusion in the study.

Variable	eIPOS patients *n* = 82
	*N* (%)
Age	
Mean (SD)	68.5 (12.1)
Median (Range)	70 (23-99)
≦69	37 (45.1)
70–79	31 (37.8)
80–89	13 (15.9)
≧90	1 (1.2)
Gender	
Male	42 (51.2)
Female	40 (48.8)
Survival of the study period	75 (92.6)
Palliative phase	
Stable	24 (32.0)
Unstable	27 (36.0)
Deteriorating	24 (32.0)
Terminal	0
Diagnoses (multiple answers possible):	
Malignant tumours/cancer	71 (86.5)
Cardiovascular disease	22 (28.2)
Neurological disease	15 (18.9)
Renal disease	6 (7.3)
Respiratory disease	22 (28.2)
Australian Karnofsky performance status	
30%	7 (8.5)
40%	8 (9.8)
50%	29 (35.4)
60%	15 (18.3)
70%	11 (13.4)
80%	5 (6.1)
Missing	7 (8.5)

### Technical feasibility

Each participant received one introductory and one final visit. Additionally, the study nurses attempted to contact participants once during the study period to ensure that study procedures were followed adequately. The introductory visit, during which the study and the eIPOS were explained, lasted on average 46 min (range 5–95 min). Due to pandemic-related restrictions, 13 of these visits were conducted by telephone. The final visit lasted on average 28 min (range 10–70 min), with 23 conducted by telephone. A total of 9 patients encountered technical difficulties during the initial visit: eight patients did not have internet access and one patient did not wish to use internet-based tools. Although study nurses attempted to clarify internet availability during the initial telephone contact, this was not always feasible. When the patients were asked how often they would like to complete the eIPOS, most participants preferred to complete the eIPOS 2–3 times per week (n = 58/82, 71%), followed by 4–5 (n = 14/82, 17%), and 6 times per week (n = 4/82, 5%). Three participants indicated that they would send the eIPOS daily, whereas information was missing for three patients. On average, the actual completion frequency aligned with the preference of most patients, namely 2–3 times per week. The care teams received a total of 470 eIPOS forms, averaging 5.7 forms per patient. Three patients did not submit any eIPOS, while one submitted 15 forms. The number of missing IPOS-items for the closed-ended questions was low. The least missing responses were for the question about “being affected by drowsiness” (n = 1), while the most were for the question about “practical problems” (n = 25). The open-ended questions had lower response rates. The non-response rates in the section where participants optionally could list and rate possible additional symptoms ranged from 48% to 90%. See Table 2. Of the 470 eIPOS forms, 286 (61%) were completed by the patients themselves, 44 (9%) with assistance from the home care team, 123 (26%) with support from relatives, in 17 cases (4%), the mode of completion was not reported.

**Table 2.** Not reported items from the 470 eIPOS forms submitted.

Questions	*n*	%
Q1. What have been your main problems or concerns over the past 3 days?		
Main problem or concern (I)	107	22.8
Main problem or concern (II)	248	52.8
Main problem or concern (III)	341	72.6
Q2. Below is a list of symptoms, which you may or may not have experienced. For each symptom, please tick one box that best describes how it has affected you over the past 3 days.		
Pain	4	0.9
Shortness of breath	5	1.1
Weakness or lack of energy	7	1.5
Nausea (feeling like you are going to be sick)	8	1.7
Vomiting (being sick)	8	1.7
Poor appetite	9	1.9
Constipation	4	0.9
Sore or dry mouth	6	1.3
Drowsiness	1	0.2
Poor mobility	5	1.1
Please list any other symptoms not mentioned above, and tick one box to show how they have affected you over the past 3 days.		
Other symptom 1	224	47.7
Affected by other symptom 1	240	51.1
Other symptom 2	332	70.6
Affected by other symptom 2	340	72.3
Other symptom 3	416	88.5
Affected by other symptom 3	424	90.2
Q3. Have you been feeling anxious or worried about your illness or treatment?	6	1.3
Q4. Have any of your family or friends been anxious or worried about you?	15	3.2
Q5. Have you been feeling depressed?	8	1.7
Q6. Have you felt at peace?	22	4.7
Q7. Have you been able to share how you are feeling with your family or friends as much as you wanted?	8	1.7
Q8. Have you had as much information as you wanted?	11	2.3
Q9. Have any practical problems resulting from your illness been addressed? (such as financial or personal)	25	5.3
Q10. How did you complete this questionnaire?	17	3.6

### Understanding the feasibility of eIPOS: results of the focus groups

We conducted three focus groups with 11 participants in total (4/3/4) (study nurses and healthcare professionals, both with experience using eIPOS in clinical care and/or providing the intervention to patients; median duration: 73 min). To ensure confidentiality of the small and specific sample, we do not publish any further details about focus group participants. The analysis of the focus groups resulted in five thematic framework categories: *Recruitment process, Patients’ condition during recruitment and use of eIPOS, Technical issues, Role of informal caregivers, Impact of specialist palliative home care setting teams attitude towards eIPOS (For further details about the thematic framework, see the Supplementary Materials.)*. Table 3 provides content and quotes of our focus group results.

**Table 3.** Results from the focus groups with study nurses and professionals.

Topic	Content
Facilitators and barriers in the recruitment process	Screening patients regarding inclusion criteria • Variation in the integration of study nurses within the teams, level of involvement proved crucial for effective collaboration with health care professionals during patient screening • Repeated screening was necessitated: “We usually did not screen all patients at once, but I asked the health care professional: “Do you have five minutes off right now?”. So we can quickly screen three patients or so. Well, that’s how it worked!” [study nurse] • Strict recruitment regulations and narrow inclusion criteria resulted in a limited number of eIPOS users. Focus group participants noted that patients in better health and requiring less intensive care were more likely to qualify for inclusion. Informing patients about the study • Prior information about eIPOS from the responsible health care professional or by providing a fact sheet about eIPOS facilitated successful recruitment • Due to the Covid-19 pandemic, some introductory visits had to be made by telephone. In this case, patients received the study documents in advance by post. The results show that this “pile of documents” discouraged some patients from participating in the study and therefore from using eIPOS. • Some patients were keen to take part in the study because they wanted to support their local specialist palliative home care team or research in general.
Patients’ condition during recruitment and use of eIPOS	• Most patients were unstable shortly after starting to receive specialised palliative care at home: “I noticed quickly, you actually have to wait almost a week before you even consider patients [for eIPOS use]. Because starting with specialist palliative home care, patients are mostly unstable.” • Duration of care in specialist palliative home care is typically brief, with stabilised patients often referred to generalist palliative home care and only returning to specialist palliative home care if their condition deteriorates. In such cases, participation in the study was no longer feasible. • When a patient met the inclusion criteria, the point in time at which the study information was delivered was critical. The end-of-life situation mostly meant that the patient had to deal with a lot of information at once: “Lifetime is so short for the patients. [. . .] They get a lot of information from us anyway. We babble on about who we are, what we do, have to explain everything and the abbreviation SPHC (specialist palliative home care), what is that? [. . .] So, this is so much new territory. And then [eIPOS] is still new territory. That’s what makes it difficult.” • Specialist palliative home care teams prioritised information provided to patients, often considering eIPOS less critical as it was not viewed as essential for improving the patient’s situation
Feasibility of the ePROM intervention influenced by technical issues	• Many specialist palliative home care patients did not have access to the internet or did not use the internet on their own, especially older patients • For those who have access to the internet, but are not familiar with its use, it was helpful to receive personal technical introduction to the use of eIPOS by the study nurse. As soon as the contact had to be via telephone, due to pandemic reasons, this support could not be provided and caused rejection of those patients, who did feel insecure in the use of eIPOS: “Here was the problem, that you could no longer pick up patients who are simply unsure about internet use by saying: I’ll come over and we’ll do it together. We’ll go through it together. Now they simply do not participate.” • Certain technical barriers could be mitigated by providing a user-friendly application (app) for smartphones or tablets, allowing patients to access eIPOS without the need to log in each time.
Informal carers role in the feasibility of ePROM in palliative home care	Handling informal caregivers’ support for the study • Informal caregivers were allowed to support patients to use eIPOS if they were physically unable to type the answers. Focus groups highlighted different involvement of informal caregivers in the study centres. • Some teams recruited patients not able to use eIPOS on their own as long as their family caregivers typed the information into eIPOS. Other teams excluded those patients. Informal caregivers facilitating or hindering eIPOS use • In the recruitment process, informal caregivers sometimes facilitated patients participation, in other cases they acted as gatekeepers to protect their relatives from anticipated further burden. • In some cases, where eIPOS users situations deteriorated rapidly, the support of informal caregivers helped them continue to report their palliative symptom burden and concerns electronically through eIPOS.
Impact of specialist palliative home care teams attitude towards PROMs and eIPOS	• The attitude of the teams towards (digital) patient-reported outcome measurement also emerged as a topic with a strong impact on the recruitment process • In cases when the responsible health care professional had introduced eIPOS before the the study nurse called, patients tended to participate more readily: “All of the patients for whom our team had announced [the study] participated.” • Regardless, it remained challenging to integrate eIPOS use into the team’s routine: “It was difficult to integrate it into the team [. . .]. You had to keep reminding everyone: remember, folks!” • Specialist palliative home care teams were initially reluctant to participate in the study, feeling that other aspects of care were more important and that the technical focus was unnecessary. • Further negative attitudes towards the study included holding views that a technical focus was not compatible with palliative care principles and other aspects of care taking precedence over symptom assessment, in particular building intensive relationships with the patients and their families. However, many professionals reported a change in their attitude following positive experiences with eIPOS during the study, leading to a more open and accepting perspective.

### Interweaving quantitative and qualitative findings: Integrated Results

**Figure 2.**
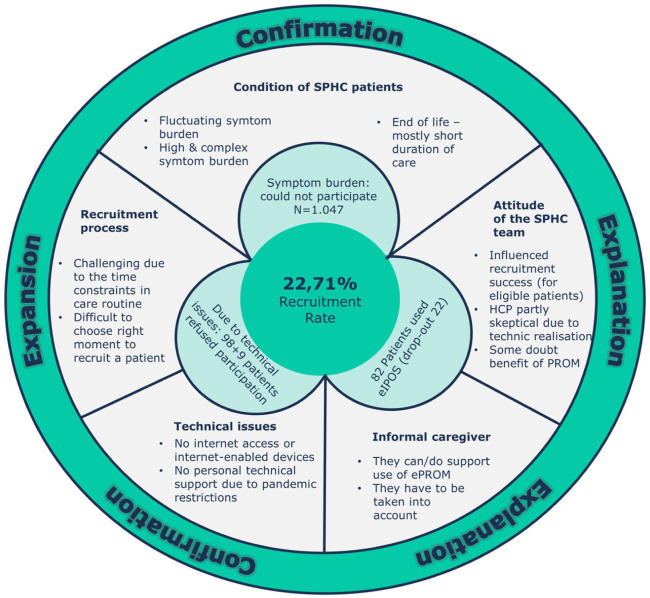
Data integration.

All thematic categories of the qualitative results were integrated with the quantitative results to provide a comprehensive picture of the feasibility of ePROM use in specialist palliative home care, as shown in Figure 2. The integration process showed that our findings confirmed, explained and even expanded each other’s understanding of our study objectives. Only 22.7% of patients who met the inclusion criteria used eIPOS during the study. The attitude of the home care teams had a significant impact on recruitment success: when health care professionals actively promoted eIPOS as a tool to support care, more eligible patients chose to participate. Conversely, some teams were reluctant to promote its use, citing scepticism about its benefits and concerns about integrating technology into palliative care. Qualitative insights into recruitment challenges provided a deeper understanding of the recruitment rate achieved. Although informal caregivers were not a formal part of the study design, they played a crucial role in hindering or facilitating the use of eIPOS. For example, they supported the use of eIPOS by providing technical support or by continuing to use it when the patient’s condition deteriorated considerably. Our findings on the condition of specialist palliative home care patients further confirmed qualitative data showing that many patients were unable to use ePROM due to their symptom burden. Technical barriers were another significant factor preventing participation, even among patients who met the inclusion criteria. Qualitative data elaborated on these barriers, highlighting issues such as lack of internet-connected devices or insufficient personal technical support due to pandemic-related contact restrictions.

## Discussion

Our study provides insights into the feasibility of ePROM in specialist palliative home care, focusing especially on the use in severely ill patients and the challenges of recruitment in this setting. Initiated in 2019, our study was among the first to use an ePROM in a palliative home care setting that patients could complete on their own devices, with data directly integrated into the electronic patient record. This eliminated the need for care teams to access an additional system. Although interest in ePROM in palliative care has grown, other studies often provide patients with internet-enabled devices^30,31^ or store ePROM data in external systems, rather than integrating it directly into the patient record.^32–34^ We did not direct patients when to use eIPOS but encouraged them to integrate the eIPOS use into their daily routines without fixed completion times or limits. Study nurses provided guidance mainly during the initial setup. Prior to our feasibility study, non-digital PROMs were rarely used in the participating study centres, so the results may also provide information on the general feasibility of PROMs in a specialist palliative home care setting.

We did not reach the target number of eIPOS users in the intervention group because of the following reasons: First, the COVID-19 pandemic disrupted recruitment, as restrictions limited home visits and shifted communication to phone and mail, which may have overwhelmed or deterred patients. Second, the short duration of care in specialist palliative home care, often due to very late admission, left little opportunity for study participation, with 80% of patients excluded due to poor health or cognitive impairment.

### Recruitment

Patients receiving palliative care show a high level of interest (69.0%) and a positive attitude (75.9%) towards digital health technologies.^35^ While the use of ePROM in clinical settings has been evaluated as feasible and acceptable, remote completion from home appears to be more difficult.^36,37^ Other studies on ePROMs in various oncological or palliative care settings reported higher recruitment rates from about 60% to 94%.^31,34,38,39^ Higher recruitment rates may be due to patients being younger and in earlier stages of the disease. Physical health status, such as lower Karnofsky status, older age and shorter survival, has been identified as a factor negatively influencing participation and use of ePROM systems.^9^ Also patients with significant fatigue showed lower compliance in completing ePROMs.^40^ In a study involving older men with advanced prostate cancer, only 20% of participants successfully completed ePROM remotely from home.^36^ Another potential factor influencing the recruitment rate in some studies was that participants were recruited in the waiting areas of clinics.^30,41^ Thus, patients did not have to overcome technical problems themselves and were potentially also fitter and earlier in their disease trajectory. The literature highlights that patients are better prepared and more willing to participate if they can discuss the ePROM process with an assistant who can dedicate more time to their questions than clinicians during standard in-hospital consultation.^42^ In our study, this crucial support was provided with varying degrees of intensity due to the emerging COVID-19 restrictions. While some participants received comprehensive initial assistance from study nurses, others were limited to telephone support. Recruitment in our defined study population was challenging, as patients at the highest level of care were often unstable and deteriorated quickly. Study nurses had to wait at least 1 week for stabilisation before introducing the study and had to screen repeatedly. Studies like MyPal and RELIEF demonstrated higher recruitment rates when targeting patients at earlier disease stages or providing onsite support and app-based systems.^40,43^ We therefore suggest to implement ePROM in an earlier stage of palliative home care, when patients condition is better and more stable. When ePROMs are introduced, for example, in generalist palliative care, lower care levels in specialist palliative home care and earlier in the disease trajectory, changing symptom burden and problems would be detected earlier and patients would be fitter to get familiar with the digital use which might be helpful when they deteriorate. Additionally, offering user-friendly, app-based ePROM platforms and ensuring robust technical support could mitigate barriers encountered in this study.

### Missing Data

In line with previous reviews that reported missing data and dropouts as common challenges in ePROM studies,^44^ most dropouts in our study occurred due to deterioration or death. The rate of missing items in our study was low, particularly for closed questions. However, non-response to open-ended eIPOS items, particularly in the section related to additional symptoms, was higher compared to closed questions. This potentially reflects barriers related to patient burden, for example, to type something in the device rather than tick a box to a predefined answer or patients perceive additional items as irrelevant. However, as these open-ended questions provide extra individual information of patients, future digital tools should implement features to mitigate this burden and promote engagement. Design solutions could include voice-to-text functionality to eliminate manual typing or minimising the required input through auto-fill. The overall layout and user interface design of the digital tool is critical in this respect.

### Challenges with digital devices

Lack of familiarity with apps or internet-enabled devices remained a major barrier, as also reported in previous studies where patients declined participation due to technical challenges.^43,45^ Other technical obstacles mentioned in previous studies include registration emails being filtered into spam folders.^36^ In order to increase acceptance, the technology should be user-friendly and intuitive.^36,37,46^ This finding was consistent with insights from our focus group, where participants underlined the importance of simplicity and ease of use for successful implementation. To address technical challenges, numerous studies provided personal assistance with the use of ePROMs or offered direct assistance during hospital visits when issues arose.^40^ As a consequence of social and technological transformation, future patients will be much more familiar with digital use and handling of devices and some of the actually observed barriers may not be relevant in future ePROM utilisation.

### Role of informal carer

In this context, informal carers can play a key role in supporting patients who are unsure about using digital tools. In our study, informal carers helped patients by either reading aloud or typing information into eIPOS on their behalf. Nevertheless, the answers were reported from the patients themselves but we cannot rule out that this assistance could have influenced the content of the ePROM responses. Providing tailored training on the purpose and use of PROMs to informal carers can help address their uncertainties, empowering them to effectively support patients. Future studies should therefore consider actively involving informal carer in the implementation of ePROMs.

### Attitudes of professionals towards ePROMs

Our results indicate that the attitude of the care team towards eIPOS significantly influenced the recruitment process. In a Canadian study nurses were less supportive of the ePROM study when they perceived limited benefit for patients.^46^ In our study, some healthcare professionals were sceptical about the suitability of eIPOS for the patient group involved, which impacted recruitment efforts. Notably, most teams in our study used structured patient-reported outcome measures for the first time, which may have influenced the feasibility outcomes of digital PROMs. However, after using the eIPOS, healthcare professionals reported that it is an important tool to support clinical routine. Most healthcare professionals indicated that the eIPOS helped them to better identify patients’ symptom burden and that the information provided via eIPOS was useful for their clinical work.^16^

### Strengths & limitations

A key strength of the study is that participating specialist palliative home care teams had little or no prior experience with PROMs, yet were able to use a digital version (ePROM) in routine care - demonstrating the feasibility for a special group of patients even in settings with limited prior exposure. The inclusion of teams from both urban and rural settings is a further strength showing that digitalisation has arrived irrespective of regional differences. However, there are also a number of limitations which limit the generalisability of the study. The originally planned quasi-experimental design could not be maintained due to the poor quality of control group data. This limits the internal validity of the study and precludes direct causal inferences. However, the descriptive design still provides valuable insights into the feasibility and acceptability of eIPOS in a real-world specialist palliative home care setting. Furthermore, the recruitment rate does not meet classic feasibility benchmarks from more than 50%. Reasons were the highly selective inclusion criteria which allowed only patients receiving the most intensive care levels in specialist palliative home care, including 24/7 access to the on-call system, to participate in the study. Furthermore, recruitment and data collection were severely affected by the COVID pandemic which reduced patients’ willingness to participate in the study and limited face-to-face patient contact. To account for these two aspects we replaced the initial visit by the study nurses with a telephone call which allowed the study nurses to introduce the eIPOS to patients over the phone. We also extended the recruitment period to gain additional time to reach potential participants. Limitations also include potential bias in focus group results due to selective sampling, recruitment challenges from high staff workloads, and variability in organisational structures across centres. To reduce potential bias, two focus groups with healthcare professionals were conducted on different days and at varying times to facilitate participation of staff with different schedules and to enhance diversity in group composition.

## Conclusion

ePROM provide a valuable resource for more patient-centred care. Their use should be considered for patients earlier in their disease trajectory, as they are still in better health and probably more open to the introduction of new ways of patient empowerment. Although digital literacy remains a challenge for older patients, this is likely to change in the future. The implementation in specialist palliative home care is generally feasible, but the complex situation of patients, their short life expectancy, and technical challenges limit their use, especially in the group of patients receiving the highest level of care. Informal carers could also be introduced to support patients or provide proxy assessments information on patients when they are too ill. Organisational readiness is crucial for the integration of PROMS and thus of ePROMs in clinical care. Accordingly, strategies are necessary for the successful implementation of PROMs.

## Supplemental Material

sj-pdf-1-pmj-10.1177_02692163251409294 – Supplemental material for Digital patient-reported outcome measures in palliative home care: A feasibility study Supplemental material, sj-pdf-1-pmj-10.1177_02692163251409294 for Digital patient-reported outcome measures in palliative home care: A feasibility study by Katerina Hriskova, Isabel Sophie Burner-Fritsch, Farina Hodiamont, Anna Bolzani, Stefanie Kolmhuber, Christina Ramsenthaler and Claudia Bausewein in Palliative Medicine
