# Supplementary material for: Digital patient-reported outcome measures in palliative home care: A feasibility study
Source: Palliat Med. 2026 Feb 5;40(5):632–43. doi: 10.1177/02692163251409294 (PMC13136539; doi:10.1177/02692163251409294)
Supplement: sj-pdf-1-pmj-10.1177_02692163251409294 – Supplemental material for Digital patient-reported outcome measures in palliative home care: A feasibility study [file sj-pdf-1-pmj-10.1177_02692163251409294.pdf]

Hriskova/Burner-Fritsch: “Digital patient reported outcome measures in palliative home care” – coding framework

Main Theme	Subtheme	Summary of Content
**1. Facilitators and barriers in the recruitment process**	Screening process	Data sequences containing information about process of screening the patients regarding the inclusion criteria: how where the inclusion criteria checked; to which point of time did the screening take place in the clinical care routine and in the patients` individual care process; how did the collaboration between the Study Nurse and the SPHC team in screening process work
	Informing patients about the study	Data sequences containing information about how eligible patients were addressed: how were patients informed about the study; did they receive information about eIPOS before the initial contact with the Study Nurse; mode of introductory visit (personal/telephonic); patients’ willingness to participate
**2. Patients’ condition during recruitment and use of eIPOS**		Data sequences containing information about patients’ condition in the process of using eIPOS or recruiting them to participate in the study: (changing) patients’ condition and related level of specialist palliative home care intensity in correlation with study participation and eIPOS use; priorisation of topics and content in patient information due to high symptom burden
**3. Feasibility of the ePROM intervention influenced by technical issues**		Data sequences containing information about technical issues facilitating or hindering feasibility of eIPOS use for patients: digital literacy; technical barriers due to eIPOS technical design; technical introduction in eIPOS use; user friendliness; ideas for technical improvement
**4. Informal caregivers’ role in feasibility of ePROM in palliative home care**	Caregiver involvement policies	Data sequences containing information about how the involvement policies for informal care giver in eIPOS use as formulated in the study protocol were handled in the study centres;
	Caregivers as facilitators or barriers	Data sequences containing information about the role of informal care givers in facilitating or hindering study participation and eIPOS use.
**5. Impact of SPHC teams’ attitude towards PROMs and eIPOS**		Data sequences containing information about the SPHC team members`attitude towards digital patient-reported outcome measurement and the feasibility study; level of engagement towards the study;
